# Integrated optoelectronic chip technologies for quantum information processing

**DOI:** 10.1093/nsr/nwag598

**Published:** 2026-09-15

**Authors:** Lantian Feng, Kun Wang, Linrunde Tao, Yun Zheng, Xinyu Jia, Nachuan Li, Bohao Zhang, Zhenda Xie, Yan-Xiao Gong, Ping Xu, Jianwei Wang, Xifeng Ren, Qihuang Gong, Shining Zhu, Guangcan Guo

**Affiliations:** Laboratory of Quantum Information, University of Science and Technology of China, Hefei 230026, China; CAS Synergetic Innovation Center of Quantum Information & Quantum Physics, University of Science and Technology of China, Hefei 230026, China; Anhui Province Key Laboratory of Quantum Network, University of Science and Technology of China, Hefei 230026, China; Hefei National Laboratory, Hefei 230088, China; College of Computer Science and Technology, National University of Defense Technology, Changsha 410073, China; National Laboratory of Solid State Microstructure, School of Physics, School of Electronic Science and Engineering, and Collaborative Innovation Center of Advanced Microstructures, Nanjing University, Nanjing 210093, China; State Key Laboratory for Mesoscopic Physics, School of Physics, Peking University, Beijing 100871, China; State Key Laboratory for Mesoscopic Physics, School of Physics, Peking University, Beijing 100871, China; National Laboratory of Solid State Microstructure, School of Physics, School of Electronic Science and Engineering, and Collaborative Innovation Center of Advanced Microstructures, Nanjing University, Nanjing 210093, China; Laboratory of Quantum Information, University of Science and Technology of China, Hefei 230026, China; CAS Synergetic Innovation Center of Quantum Information & Quantum Physics, University of Science and Technology of China, Hefei 230026, China; Anhui Province Key Laboratory of Quantum Network, University of Science and Technology of China, Hefei 230026, China; National Laboratory of Solid State Microstructure, School of Physics, School of Electronic Science and Engineering, and Collaborative Innovation Center of Advanced Microstructures, Nanjing University, Nanjing 210093, China; Hefei National Laboratory, Hefei 230088, China; National Laboratory of Solid State Microstructure, School of Physics, School of Electronic Science and Engineering, and Collaborative Innovation Center of Advanced Microstructures, Nanjing University, Nanjing 210093, China; Hefei National Laboratory, Hefei 230088, China; College of Computer Science and Technology, National University of Defense Technology, Changsha 410073, China; Hefei National Laboratory, Hefei 230088, China; State Key Laboratory for Mesoscopic Physics, School of Physics, Peking University, Beijing 100871, China; Laboratory of Quantum Information, University of Science and Technology of China, Hefei 230026, China; CAS Synergetic Innovation Center of Quantum Information & Quantum Physics, University of Science and Technology of China, Hefei 230026, China; Anhui Province Key Laboratory of Quantum Network, University of Science and Technology of China, Hefei 230026, China; Hefei National Laboratory, Hefei 230088, China; Hefei National Laboratory, Hefei 230088, China; State Key Laboratory for Mesoscopic Physics, School of Physics, Peking University, Beijing 100871, China; National Laboratory of Solid State Microstructure, School of Physics, School of Electronic Science and Engineering, and Collaborative Innovation Center of Advanced Microstructures, Nanjing University, Nanjing 210093, China; Laboratory of Quantum Information, University of Science and Technology of China, Hefei 230026, China; CAS Synergetic Innovation Center of Quantum Information & Quantum Physics, University of Science and Technology of China, Hefei 230026, China; Anhui Province Key Laboratory of Quantum Network, University of Science and Technology of China, Hefei 230026, China; Hefei National Laboratory, Hefei 230088, China

**Keywords:** optoelectronic integration, photonic quantum technology, quantum information processing, chip technology

## Abstract

In photonic quantum information processing, the generation, manipulation, and detection of photonic quantum states inherently depend on electronic control devices. As a core enabling technology, on-chip optoelectronic hybrid integration drives optical quantum devices toward higher density, lower power consumption, and enhanced stability while pushing the application and performance limits of quantum information processing. This review summarizes the progress in chip-integrated optoelectronic technologies and their applications in key quantum information scenarios—quantum communication, quantum photonic source engineering, optical frequency conversion, and linear optical quantum information processing. We also discuss key challenges and future directions, envisioning optoelectronic integration as the pivotal bridge from lab demonstrations to practical, scalable quantum photonic systems.

## Teaser

Heterogeneous integration enables scalable and manufacturable quantum photonic systems. Here, we review the latest key optoelectronic chip progress and outstanding challenges for full scale quantum information processing.

## INTRODUCTION

Rapid advances in integrated optoelectronic chip technologies have revolutionized photonic systems by replacing bulky discrete assemblies with compact, scalable integrated chips. This enables precise control over light generation, modulation, and detection, driving breakthroughs in classical optical technologies including high-speed modulators [1,2], optical frequency combs [3], and high-sensitivity optical field detection [4]. In recent years, these capabilities have been extended to the quantum regime, enabling new approaches to manipulating nonclassical light—such as quantum light sources [5–7], photonic quantum circuits [8–10], and single-photon detectors [11,12]. With breakthroughs in heterogeneous integration for these complementary components, a complete set of integrated devices has been developed for scalable, manufacturable quantum photonic systems. These integrated platforms facilitate the advancement of quantum information processing applications, including quantum communication [13,14], quantum computing [15], and quantum interfaces [16].

In this review, we cover the latest key developments in optoelectronic integrated chip technology and the remaining challenges for fully integrated quantum chips in quantum information processing. The review is structured as follows: Section ‘OPTOELECTRONIC INTERACTIONS AND HETEROGENEOUS INTEGRATION’ discusses fundamental optoelectronic interactions and heterogeneous integration strategies to overcome material limits. Section ‘BUILDING OPTOELECTRONIC BLOCKS FOR INTEGRATED QUANTUM CHIPS’ focuses on

core optoelectronic building blocks for integrated quantum chips. Section ‘APPLICATIONS OF INTEGRATED OPTOELECTRONIC QUANTUM CHIPS’ highlights cutting-edge quantum information applications. Finally, conclusions and outlook are provided in Section ‘OUTLOOK’.

## OPTOELECTRONIC INTERACTIONS AND HETEROGENEOUS INTEGRATION

Photonic quantum chips rely on three core functionalities: nonclassical photon generation, quantum state manipulation, and single-photon detection. In photon generation stage, nonclassical light is produced via nonlinear optical effects or spontaneous radiative transitions in solid-state emitters. This process is fundamentally determined by the intrinsic electronic properties of materials, and its performance is primarily optimized through device-level engineering strategies, such as resonant enhancement enabled by optical microcavities. Therefore, this research direction is not the focus of the present review, relevant in-depth discussions can be found in existing review articles [17,18]. Differently, a variety of optoelectronic integration technologies are extensively adopted for quantum state regulation and single-photon detection, which will be introduced and reviewed in sequence.

### Optoelectronic interactions in quantum state preparation

In quantum state preparation stage, linear optical strategies such as spectral filtering, polarization manipulation, and mode engineering are adopted to regulate photonic degrees of freedom and construct quantum states. We first briefly introduce and review the key optoelectronic integration technologies employed in this stage.

#### Thermo-optic effect

The thermo-optic effect utilizes temperature variations to induce corresponding changes in the refractive index of a material. To control the phase of the target structure, resistive heaters are precisely integrated at different positions on the chip to locally control the temperature. The phase shift generated by the resistive heater can be formulated as [19]


(1) \begin{eqnarray*}
\Delta {\varphi }=\frac{2\pi }{\lambda }\left(\frac{\mathrm{d}n_{\mathrm{eff}}}{\mathrm{d}T}\right)\Delta {T}L,
\end{eqnarray*}

where $n_{\mathrm{eff}}$ denotes the effective refractive index, *λ* is the wavelength of light, and *L* is the length of the resistive heater. The temperature change $\Delta {T}$ is proportional to the power consumed by the resistive heater *P*, and this relationship can be mathematically expressed as *Δ T = kP*. Here, the proportionality coefficient *k* is associated with the thermal properties of the material, the structural parameters of the device, and other environmental or systemic factors. Thus, the achieved phase $\Delta {\varphi }$ is proportional to the power dissipated by the resistor. This results in a quadratic relationship between the applied current of the heater and the induced phase shift, that is, $\Delta {\varphi (I)}=\alpha +\beta {I^2}$.

Common photonic devices that utilize thermo-optic phase shifters are thermo-optic modulators (TOMs), with typical configurations being Mach–Zehnder interferometers and microring resonators. TOMs have been widely adopted in quantum photonic integrated chips, owing to their inherent compatibility with scalable fabrication processes, straightforward design, cost-effectiveness, compact footprint, and flexible tuning capabilities. However, they rely on continuous Joule heating to achieve phase tuning, with a typical half-wave power consumption of $P_\pi =20-50$ mW, resulting in large static power consumption and severe thermal crosstalk. Improving the tuning efficiency and reducing thermal crosstalk are critical to enhancing the performance. For the latest advances, see the review by Liu *et al*. [20].

#### Pockels electro-optic effect

Let $\mathbf {E}=(E_x,E_y,E_z)$ be the externally applied electric field in the crystal principal-axis coordinate system, with $E_x$, $E_y$, and $E_z$ denoting its components along the *x*-, *y*-, and *z*-axes, respectively. The Pockels electro-optic (EO) effect describes the linear change in the refractive index of a crystal induced by an externally applied electric field, typically characterized by the linear EO tensor:


(2) \begin{eqnarray*}
\left[{\begin{array}{c}
\Delta \bigl (\frac{1}{n^2}\bigl )_1 \\
\Delta \bigl (\frac{1}{n^2}\bigl )_2 \\
\Delta \bigl (\frac{1}{n^2}\bigl )_3 \\
\Delta \bigl (\frac{1}{n^2}\bigl )_4 \\
\Delta \bigl (\frac{1}{n^2}\bigl )_5 \\
\Delta \bigl (\frac{1}{n^2}\bigl )_6 \end{array}}\right]
= \left[ {\begin{array}{c@{\quad}c@{\quad}c}
r_{11} & r_{12} & r_{13} \\
r_{21} & r_{22} & r_{23} \\
r_{31} & r_{32} & r_{33} \\
r_{41} & r_{42} & r_{43} \\
r_{51} & r_{52} & r_{53} \\
r_{61} & r_{62} & r_{63} \end{array}}\right]
\left[ {\begin{array}{c}
E_x \\
E_y \\
E_z \end{array}}\right],
\end{eqnarray*}

where $r_{ij}$ are the linear EO coefficients and $\Delta (1/n^2 )_i=\sum _{j=1}^{3}r_{ij}E_j$ denotes the field-induced perturbation of the *i*th contracted component of the optical impermeability tensor [21]. Here, $E_1$, $E_2$, and $E_3$ correspond to $E_x$, $E_y$, and $E_z$, respectively.

For a uniaxial crystal (e.g. lithium niobate, point group 3 m), most EO coefficients are zero, and the non-zero EO coefficients satisfy $r_{13}=r_{23}, r_{33}, r_{22}=-r_{12}, r_{51}=r_{42}$ [22]. When the optical field is polarized along the extraordinary (*e*) axis and the electric field is applied along the *z* axis, the dominant coefficient $r_{33} \approx$ 30.8 pm/V determines the modulation strength. The variation of the extraordinary refractive index is


(3) \begin{eqnarray*}
\Delta n_e = -\frac{1}{2}n_e^3 r_{33}E_z,
\end{eqnarray*}

where $n_e$ is the extraordinary refractive index and $\Delta n_e$ is its field-induced perturbation. For a guided mode, the effective index change can be approximated as $\Delta n_{\mathrm{eff}}\simeq \Gamma \Delta n_e$, where *Γ* is the electro-optic overlap factor.

As a consequence, light propagating through an EO crystal undergoes a phase shift arising from the intrinsic refractive index of the crystal, plus an additional phase shift $\Delta \varphi$ proportional to the effective index change $\Delta n_{\mathrm{eff}}$ and the propagation length *L*. Based on Equation (3), the phase shift for *y*-propagating TE$_{00}$ light in a *x*-cut thin-film lithium niobate (TFLN) waveguide with an electric field along the optical *z*-axis can be expressed as


(4) \begin{eqnarray*}
\Delta \varphi = \frac{2\pi \Delta n_{\rm eff}}{\lambda }L = -\frac{2\pi }{\lambda }\frac{1}{2}n_e^3 r_{33} E_z\Gamma L =-\pi \frac{V}{V_\pi }.\!\!\!\! \\
\end{eqnarray*}

In Equation (4), $V={E_z}{d}$ denotes the applied voltage, where *d* is the gap between the two electrodes applying the voltage. $V_\pi ={\lambda d}/{n_e^3 r_{33} \Gamma L}$ represents the voltage required to achieve a *π* phase shift.

Benefiting from intrinsic ultra-fast lattice polarization, the EO effect shows a femtosecond-to-picosecond response. However, unlike the thermo-optic effect (broadly material-agnostic), the linear EO effect is forbidden in centrosymmetric media (e.g. silicon). Beyond LN, promising EO materials including lithium tantalate [23], barium titanate [15], lead zirconate titanate [24], and aluminum nitride [25] also exhibit excellent performance. For large-scale quantum applications, on-chip EO modulator integration scale requires further improvement, while fabrication, device dimensions, and packaging/measurement-control capabilities are advancing rapidly.

#### Free-carrier dispersion effect

For high-speed optical modulation in silicon photonics, the free-carrier dispersion effect is widely adopted. It relies on a key mechanism: free-carrier density variations in silicon alter its refractive index and absorption coefficient. Compatible with complementary metal-oxide-semiconductor (CMOS) processing, the free-carrier dispersion effect has become the foundation of state-of-the-art silicon modulators. Most recent high-performance devices use this effect, with the free-carrier depletion in rib waveguides being the dominant approach [26]. Despite their advantages, free-carrier dispersion-based modulators suffer from free-carrier absorption loss and thermal sensitivity. In terms of power consumption, these devices incur both static power dissipation and dynamic switching energy. These challenges remain key issues that need to be addressed for large-scale quantum photonic applications. A detailed overview of the performance metrics of different silicon photonic modulators can be found in Ref. [27].

#### Stark effect

When manipulating microscopic quantum systems such as atoms, quantum dots, and color centers on an integrated optical quantum chip, the Stark effect is typically employed. By applying an external electric field, the energy levels of these quantum systems will shift or split, thereby enabling the controllable tuning of the light transition frequency. This energy modulation of emission can be used to regulate deterministic quantum light sources and achieve wavelength matching between different emitters.

#### Others

In addition to the above three optoelectronic interactions, other light-field modulation schemes (magneto-optic, acousto-optic, and piezoelectric effects) do not enable direct electrical control of optical signals. Instead, they rely on external electronic drivers to generate magnetic/acoustic fields or induce mechanical deformation, which in turn mediate light manipulation–forming system-level optoelectronic hybrid systems. Driven by advances in integrated photonic chip technologies, these effects have moved from discrete components to integrated optical chips, providing complementary functions for specialized quantum photonic applications such as nonreciprocal isolation [28,29] and sensing [30].

### Optoelectronic interactions in single-photon detection

Single-photon detectors convert incident photons into electrical signals through photon–matter interactions, and different types of devices rely on distinct physical principles. Two dominant working mechanisms are introduced in the following.

#### Avalanche effect

In semiconductor single-photon detectors, the avalanche effect serves as the core amplification mechanism. A high reverse bias is applied to the PN junction of the photodiode, generating a strong electric field within the depletion region. Incident single photons excite electron–hole pairs, which are rapidly accelerated by the intense electric field to gain sufficient kinetic energy. These high-energy carriers trigger impact ionization with the lattice, continuously generating new electron–hole pairs and inducing chain carrier multiplication, which eventually leads to avalanche breakdown. Operating in Geiger mode, the device amplifies extremely weak single-photon signals into detectable electrical pulses, enabling high-sensitivity single-photon detection.

#### Superconducting critical current suppression effect

In superconducting nanowire single-photon detectors (SNSPDs), the superconducting critical current suppression effect is utilized. The superconducting nanowire is biased at a current slightly below the critical current to maintain a lossless superconducting state with zero resistance. Once a single photon is absorbed, the local energy rises abruptly, generating non-equilibrium quasiparticles and a tiny resistive region that breaks local superconductivity. The emerging resistive area redirects the bias current and produces a measurable transient voltage pulse. The photon-induced local suppression of superconductivity essentially corresponds to an instantaneous reduction of the critical current, which allows the system to identify and record single-photon events. Benefiting from this mechanism, SNSPDs exhibit unique advantages including low dark count rate and ultra-low timing jitter.

For a more comprehensive understanding of single-photon detectors in quantum integrated photonics, readers may refer to the latest review article [31].

### Heterogeneous integration strategies

Monolithic integration offers a compact and fabrication-friendly route to large-scale photonic circuits, but no single material platform simultaneously optimizes all functions required for quantum photonic chips. For example, SOI offers mature foundry processing and dense passive circuitry, Si$_3$N$_4$ provides ultra-low-loss routing and delay, and TFLN enables high-speed Pockels-based electro-optic control. Heterogeneous integration therefore seeks to combine these complementary strengths with efficient emitters, detectors, and control electronics in a unified and manufacturable photonic platform.

#### Integrated sources and interfaces

A central goal is the integration of high quality emitters and quantum interfaces, often unavailable in the native photonics platform, into low loss circuits for scalable routing, multiplexing, and interference. Hybrid integration of III–V quantum dot emitters with ultra low loss silicon nitride (Si$_3$N$_4$) waveguides enables triggered single photon injection into circuits with long propagation lengths suitable for delays and large interferometers [32]. Scaling this paradigm, quantum dot microchiplets have been incorporated into foundry silicon photonic circuits with tunability, improving wavelength matching and yield in multi emitter architectures [33]. Hybrid integrated quantum network primitives that combine solid state quantum dots with low loss, reconfigurable photonic circuits further illustrate how heterogeneous integration can simultaneously deliver high quality sources and system level tunability for chip based networking and interference between independent emitters [34]. Beyond III–V emitters, direct bonding toolkits for diamond membranes provide a route to integrate color center hosts onto diverse substrates [35], while integrated molecular emitters have demonstrated high visibility on chip two photon interference between independent sources, highlighting the role of integration and tuning in achieving indistinguishability [36]. Co-integration of spin photon interfaces with cryogenic CMOS control further illustrates how heterogeneous stacks can unify quantum interfaces with local control electronics [37].

#### Passive active integration

A second direction targets the control layer by combining ultra low loss passive routing with high speed EO actuation in a single integrated stack. Wafer scale bonding of TFLN onto planarized Si$_3$N$_4$ merges Si$_3$N$_4$ class passive stability and low loss with LN class fast EO tuning, enabling reconfigurable interferometry and switching without compromising the loss budget [38], while recent back end heterogeneous integration of thin film lithium tantalate on silicon photonics further illustrates a PDK compatible route to adding high bandwidth Pockels modulation to mature passive silicon platforms [39].

#### System integration and manufacturability

The third goal moves from component integration toward platform completeness, where detection, packaging interconnects, and production facing workflows become decisive. Demonstrations co-fabricated LN nanophotonics with superconducting nanowire detectors, unifying cryogenic compatible EO reconfiguration with high efficiency single photon readout on the same circuit [40]. Integrated source stacks that combine chip excitation and nonlinear generation further reduce reliance on external subsystems, allowing more turnkey entangled qubit and qudit state generation for deployable photonic modules [41]. At the packaging level, photonic wire bonding provides a practical method to interconnect dissimilar source and circuit chips with low loss and relaxed alignment tolerances, supporting modular assembly and rework [42]. Finally, manufacturable platform efforts consolidate heterogeneous stacks and packaging into coherent scaling strategies that link component performance to standardized test flows and system assembly [15].

Overall, heterogeneous quantum photonics is evolving from isolated component-level demonstrations toward wafer-scale, PDK-compatible, and packaging-aware platforms. The main technical challenges are the co-integration of high-performance sources, ultra-low-loss interferometry, efficient detection, and fast EO control together with material/process compatibility, thermal and cryogenic co-design, interlayer coupling loss, and yield-aware testing and assembly. Converging wafer-scale heterogeneous processes with standardized design kits and packaging interfaces provides a credible route from laboratory prototypes to scalable optoelectronic quantum systems.

## BUILDING OPTOELECTRONIC BLOCKS FOR INTEGRATED QUANTUM CHIPS

This section reviews the key building of optoelectronic blocks that enable integrated quantum chips, emphasizing how integration strategies and device figures of merit translate into quantum-level performance.

### Quantum light sources

Quantum light sources are key building blocks of quantum photonic chips for computing, sensing, and networking [51]. They can be categorized along two complementary axes: by generation mechanism, as either probabilistic nonlinear sources or deterministic/near-deterministic emitter-based sources; and by information encoding scheme, as either discrete-variable (DV) sources based on photon-number, path, polarization, time-bin, or frequency-bin encoding, or continuous-variable (CV) sources such as quadrature-squeezed states. On-chip optoelectronic engineering is essential for improving source stability, spectral alignment, indistinguishability, and scalability. Next, we first review common DV sources based on different generation mechanisms, followed by a survey of emerging integrated CV sources.

#### Probabilistic quantum light sources

Probabilistic sources exploit spontaneous parametric down-conversion in $\chi ^{(2)}$ materials, such as LN [52], GaAs [53], and AlN [54], or spontaneous four-wave mixing (SFWM) in $\chi ^{(3)}$ platforms, such as silicon [16,55] and Si$_3$N$_4$ [56]. They generate photon pairs, where detection of one photon heralds its partner, and larger entangled or multi-photon states can be built through interference, post-selection, multiplexing, or fusion operations.

Straight waveguide sources, such as periodically poled LN [52] or silicon spiral waveguides [16], offer broad bandwidth and relatively high tolerance to fabrication variations. In contrast, microring resonators are widely adopted to enhance photon pair generation, improve spectral purity, and increase integration density, leveraging high intracavity intensity and a small footprint. However, their narrow resonances make their performance sensitive to fabrication errors and environmental drift, so active optoelectronic tuning is typically required. Thermo-optic tuning is widely used in silicon and Si$_3$N$_4$ microrings to align resonant wavelengths: electrical control has been applied for on-chip filtering (Fig. 1a) [43] and for generating spectrally pure and indistinguishable SFWM photons (Fig. 1b) [44,45]. Beyond direct resonance tuning, thermo-optic controlled asymmetric Mach–Zehnder interferometer couplers can reshape the coupling conditions for pump, signal, and idler modes, enabling improved spectral purity, pair generation rate, heralding efficiency, and coincidence to accidental ratio (Fig. 1c) [46,47]. For scaling to large arrays, noninvasive photocurrent sensing in tunable silicon microring cavities has been demonstrated on a commercial 45 nm CMOS platform to actively lock SFWM wavelengths and stabilize operation (Fig. 1d) [48]. Thermo-optic control also enables system level integration of pump lasers, for example using Vernier tuning in cascaded microrings on Si$_3$N$_4$ to realize an electrically pumped InP laser that supports turnkey sources of frequency bin entangled qubit and qudit states (Fig. 1e) [41].

**Figure 1.**
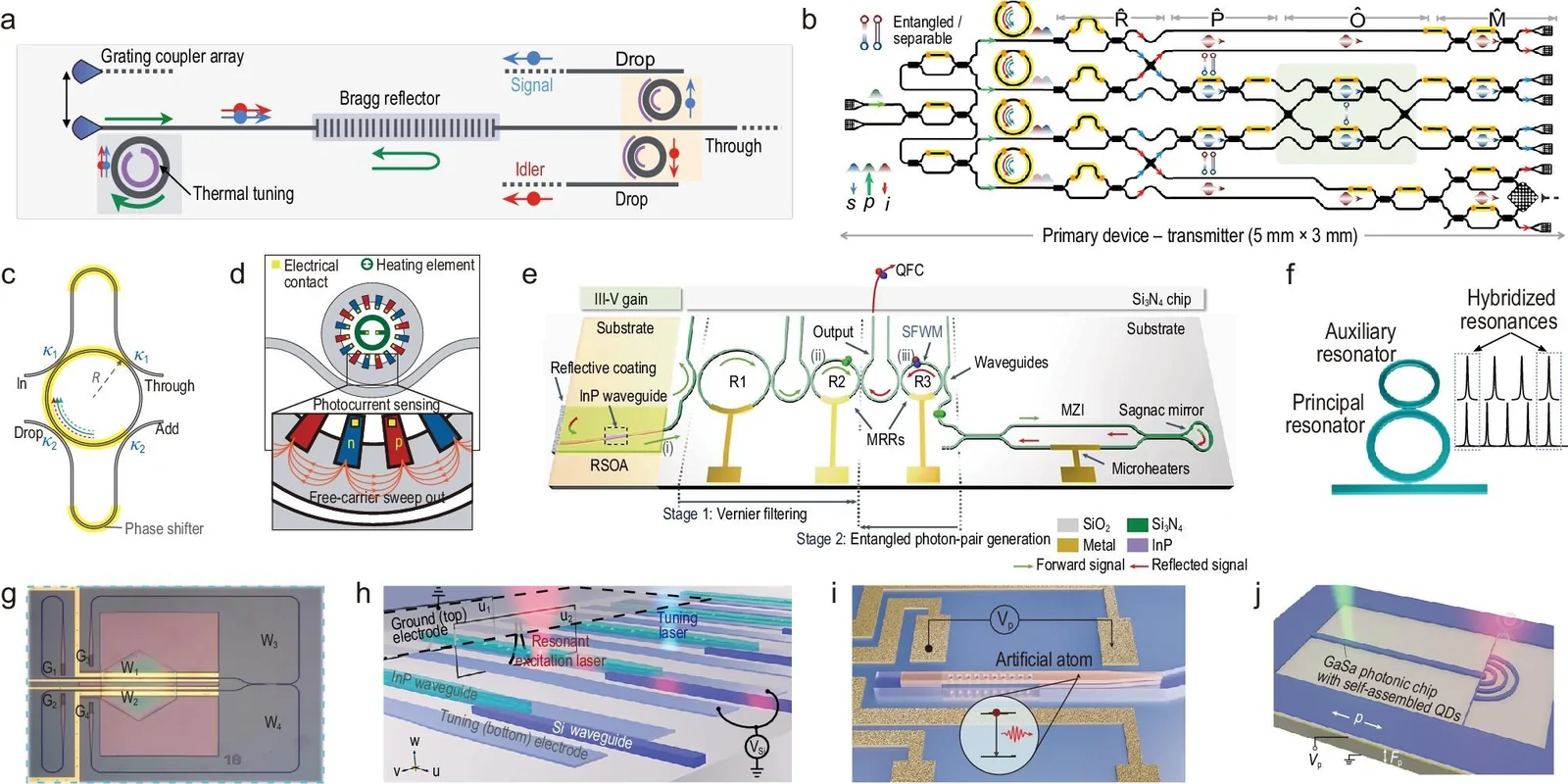
Optoelectronic engineering of integrated quantum light sources. (a–e) Probabilistic DV photon-pair sources based on tunable silicon and Si$_3$N$_4$ resonators, including on-chip filtering, spectrally pure SFWM, feedback-stabilized microrings, and turnkey frequency-bin-entangled sources [41,43–48]. (f) Integrated CV squeezed-light generation using a Si$_3$N$_4$ photonic molecule [49]. (g–j) Deterministic or near-deterministic solid-state emitters integrated with Si$_3$N$_4$, silicon, TFLN, and piezoelectric photonic platforms [33,34,36,50]. Reproduced with permissions from: (a) Ref. [43] under a CC-BY-4.0 license; (b) Ref. [45], copyright: 2020 Springer Nature; (c) Ref. [46], copyright: 2019 Optical Society of America; (d) Ref. [48], copyright: 2025 Springer Nature; (e) Ref. [41], copyright: 2023 Springer Nature; (f) Ref. [49] under a CC-BY-4.0 license; (g) Ref. [36], copyright: 2025 Springer Nature; (h) Ref. [33] under a CC-BY-4.0 license; (i) Ref. [34], copyright: 2025 Springer Nature; (j) Ref. [50], copyright: 2022 American Chemical Society.

#### Deterministic quantum light sources

Deterministic sources typically rely on solid-state quantum emitters, especially quantum dots heterogeneously integrated with photonic waveguides [57]. Compared with probabilistic sources, their key challenges are efficient photon extraction, spectral uniformity, stability, and high photon indistinguishability across many emitters. Optoelectronic engineering addresses these issues through filtering, wavelength tuning, and local stabilization. For example, tunable Si$_3$N$_4$ resonators have enabled on-chip filtering and wavelength-division multiplexing of InAsP quantum-dot photons [58]. Electric-field Stark tuning has been used to frequency-match independent molecular emitters in Si$_3$N$_4$ (Fig. 1g) [36] and InAs quantum dots integrated with silicon photonics (Fig. 1h) [33], enabling high-visibility Hong–Ou–Mandel interference. Piezoelectric strain tuning provides another route to spectral control, as demonstrated in TFLN-integrated quantum-dot devices (Fig. 1i) [34] and GaAs quantum-dot chips used for polarization-entangled photon generation (Fig. 1j) [50].

#### Continuous-variable quantum light sources

Integrated CV sources are typically generated by operating $\chi ^{(2)}$ or $\chi ^{(3)}$ nonlinear devices below threshold, producing quadrature-squeezed vacuum, two-mode squeezed states, or multimode entangled Gaussian states. In Si$_3$N$_4$, microring and coupled-resonator photonic-molecule designs have enabled broadband squeezing and suppression of parasitic nonlinear processes (Fig. 1f) [49]. In TFLN, strong $\chi ^{(2)}$ nonlinearity combined with electro-optic tunability supports compact optical-parametric-oscillator and squeezed-light devices with integrated phase control and homodyne-compatible characterization [59,60]. More recently, integrated microcombs have generated multipartite CV entanglement over multiple frequency modes [61], while monolithic CV photonic circuits have combined squeezed-light generation, qumode operations, local-oscillator preparation, and balanced homodyne detection for on-chip generation, manipulation, and verification of four-qumode cluster states [62]. A more comprehensive review of recent advances in this area can be found in the latest articles [63,64].

#### Summary of platform advantages and limitations

LN platforms offer strong $\chi ^{(2)}$ nonlinearity, efficient SPDC, and electro-optic functionality, but source performance also depends on phase matching, optical loss, pump rejection, spectral purity, fabrication repeatability, and scalability. Silicon microrings compensate weaker $\chi ^{(3)}$ nonlinearities through compact CMOS-compatible resonant enhancement, but require active stabilization and are affected by resonance drift and nonlinear absorption. Silicon spiral waveguides provide broader bandwidth and relaxed resonance control, at the cost of larger footprints and higher pump powers. Si$_3$N$_4$ offers low loss, high-*Q* resonators, and negligible telecom-band two-photon absorption, but generally requires resonant enhancement or longer interaction lengths. Thus, these platforms are complementary rather than ranked by nonlinear coefficient alone; further comparisons can be found in recent reviews on integrated nonlinear quantum light sources [65].

### Quantum state control circuits

Photonic quantum state control circuits represent the foundational hardware platform enabling the precise, dynamic, and scalable manipulation of various photonic degrees of freedom, including path, phase, polarization, time-frequency, and spatial mode. Integrated optoelectronic circuits are widely utilized to implement quantum state manipulation. Various types of quantum photonic sources encoded by different degrees of freedom [66–69], along with single-qubit gates [70–72] and multi-qubit gates [73–75], have been developed in recent years. Advances in this field of late have focused on fast reconfiguration [76], real-time feedforward [48], and programmable nonlinear circuits [77]—all of which are critical for realizing universal linear optical quantum computing and fault-tolerant photonic architectures. Some contemporary review articles [78,79] have provided a systematic overview of this rapidly evolving research area. Here, we focus specifically on the manipulation of the frequency domain through electro-optic modulation, an area that has witnessed a surge of pioneering works in recent years, and we provide a more in-depth review of this particular subfield.

In the previous section, we derived the phase shift induced by a static electric field with the Pockels EO effect. Applying a time-varying electric field (e.g. a sinusoidal microwave signal) to an EO crystal drives $\Delta \varphi$ to oscillate with the microwave amplitude, generating new optical frequencies detuned by the microwave signal [80]. This EO modulation stems from coherent microwave–optical interactions, which shift optical photon frequencies via microwave photon absorption or emission. EO modulation thus enables coherent frequency translation and mode coupling across optical channels, laying the groundwork for frequency-domain quantum information processing. In particular, the frequency domain is a powerful platform for encoding and processing of quantum information, with compatibility to fiber communications and integrated photonics, and inherent high dimensionality through multiple frequency channels [72,81,82]. In the following, we review the basic principle and typical applications of frequency control via EO modulation in quantum photonic chips.

#### Principle

The conventional formalism of EO modulation mainly involves a waveguide-based EO phase modulator driven by a modulation signal $V(t) = V_{p} \sin (\omega _m t)$, where $V_{p}$ denotes the peak voltage and $\omega _m$ is the modulation frequency [86]. When the phase modulator is illuminated by a continuous-wave laser with carrier frequency $\omega _c$ and amplitude $E_0$, the output optical field can be expressed as


(5) \begin{eqnarray*}
E_\mathrm{out}(t) = E_0 e^{i[\omega _c t + \beta \sin (\omega _m t)]},
\end{eqnarray*}

where *β* represents the modulation depth (or modulation index), defined as $\beta =\pi V_p/V_\pi$. Equation (5) can be expanded via the Jacobi–Anger expansion [87] to yield


(6) \begin{eqnarray*}
E_\mathrm{out}(t) = E_0 \sum _{n} J_n(\beta )\ e^{i(\omega _c + n\omega _m)t},
\end{eqnarray*}

where $J_n$ are the Bessel functions of the first kind. These functions characterize the amplitude of the generated sidebands, which correspond to different frequency components $\omega _n=\omega _c+n\omega _m$. This set of sidebands forms an EO frequency comb, with the integer *n* specifying the order of each comb line [88].

The conventional formalism can also be extended to the pulsed regime to realize coherent spectral shearing and frequency shifting (see the next subsection). However, this formalism is hardly applicable to complex systems where multiple frequency modes are coupled simultaneously. Instead, the mode-coupling formalism—which treats EO modulation as a coupling mechanism between frequency modes and employs the Hamiltonian formalism to describe the system in a single cavity or coupled cavities—is more appropriate.

For single-cavity-based EO modulation, two regimes should be distinguished based on the relationship among the modulation frequency ($\omega _m$), the linewidth of the resonant mode ($\delta \omega$), and the free spectral range (FSR) between adjacent resonant modes [89]:

- Sideband-resolved regime: $\delta \omega < \omega _m< < $ FSR;
- Roundtrip-resolved regime: $\omega _m\approx$ FSR or $\omega _m> $ FSR.

The unresolved regime ($\omega _m< \delta \omega$) is not considered herein, as the resonances of high-quality-factor cavities (high-Q cavities) typically exhibit megahertz-level linewidths, which are much smaller than the typical modulation frequency of several gigahertz.

In the sideband-resolved regime, the phase modulator induces frequency modulation of the form $\omega _0+\Omega \cos (\omega _m t)$ in the single cavity, corresponding to the Hamiltonian:


(7) \begin{eqnarray*}
H=\omega _0 a_0^\dagger a_0+\Omega \cos (\omega _m t)a_0^\dagger a_0,
\end{eqnarray*}

where *Ω* denotes the coupling rate induced by microwave modulation, and $a_0$ ($a_0^\dagger$) represents the annihilation (creation) operator for the resonant mode at frequency $\omega _0$. In the roundtrip-resolved regime, the roundtrip interference effect drives cavity frequency modulation, and the system Hamiltonian is given by


(8) \begin{eqnarray*}
H= \sum _{n=-N}^{N} \omega _n a_n^\dagger a_n+ \Omega \cos (\omega _m t)(a_n^\dagger a_{n+1}+h.c.),
\end{eqnarray*}

where the optical modes $a_n$ are directly modified by the EO coupling within the cavity [90].

#### Frequency shifting and splitting

Quantum spectral control entails tailoring single-photon spectra, requiring photon energy conversion without extra loss or noise. Conventional nonlinear optical approaches introduce substantial quantum noise due to high-power pumping. In contrast, EO phase modulation enables coherent deterministic spectral-temporal control via direct microwave-optical coupling. In TFLN, frequency shifting and bandwidth compression of heralded single photon pulses have been achieved using double-pass waveguide EO phase modulation (Fig. 2a, b) [83], with a record *±*641 GHz (*±*5.2 nm) frequency shift for telecom single photons. Furthermore, over 18-fold bandwidth compression was demonstrated via spectral shearing and time lensing. More recent work with an EO modulator featuring sub-1 V cm $V_\pi L$ has demonstrated spectral shearing, achieving an effective frequency shift over seven times the intrinsic pulse bandwidth across the visible-to-near-infrared range [91].

**Figure 2.**
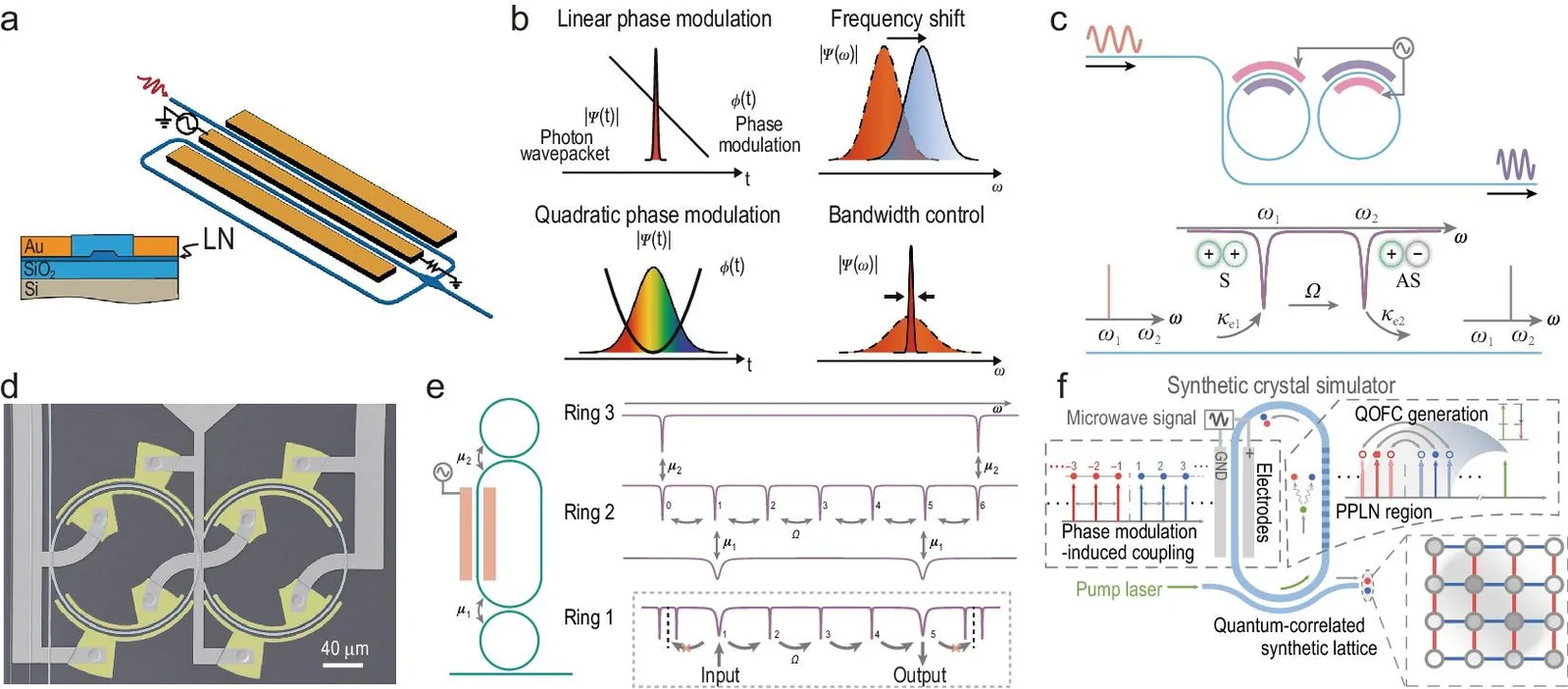
Optoelectronic devices for spectral control and frequency-bin encoding manipulation. (a) Cross-section of the TFLN EO phase modulator with the double-pass configuration. (b) Frequency and bandwidth control of light via temporal phase modulation [83]. (c) Schematic of a coupled-ring system used to implement the general critical coupling (GCC) condition (top), and its characterization in frequency domain (bottom). The coupled-ring system provides a pair of hybrid modes, referred to as symmetric (S) and anti-symmetric (AS). Coupling between them is induced by EO modulation. CW, continuous wave. (d) Scanning electron microscopy micrograph in false color of the EO frequency shifter. (e) The structure to realize cascaded frequency shifting [84]. (f) The schematic of a quantum-correlated synthetic crystal based on a TFLN racetrack resonator [85]. Reproduced with permissions from: (a, b) Ref. [83] under a CC-BY-4.0 license; (c–e) Ref. [84], copyright: 2021 Springer Nature; (f) Ref. [85], copyright: 2023 Springer Nature.

Compared to single-waveguide EO modulators, coupled-cavity architectures further advance EO-based quantum frequency control by enabling engineered multi-mode interactions and hybrid microwave-optical coupling. In a coupled-cavity system, evanescent coupling enables photon hopping and nonlinear optical interactions mediating multilevel energy transitions [84]. Such systems range from two-cavity setups to complex waveguide–resonator networks, supporting a dense lattice of optical frequency modes whose interactions are engineerable via cavity design and EO modulation [92,93]. Integrating coupled optical cavities with microwave resonators forms a microwave–optical resonant system, enabling controllable EO coupling through strong and fast EO modulation [94–96]. This system supports hybrid photonic-molecule modes [93], allowing coherent microwave-to-optical photon transduction—critical to linking microwave quantum transducers to photonic quantum networks [94,97].

Recently, high-efficiency frequency shifting up to 28 GHz with 90% on-chip conversion efficiency has been demonstrated in a two-level coupled-microring system (Fig. 2c–e) [84,98]. Under the GCC condition, high-performance EO frequency shifters and beam splitters have been realized. Cascade operation further extends the shift to 119.2 GHz using a 29.8 GHz single-tone microwave drive. These GHz-band operations are promising building blocks for large-scale integrated quantum information processing and frequency-domain photonic quantum computing. The system was also extended to single-photon level 18.2 GHz frequency shifting via coherent EO control, with HOM interference visibility of 74±0.9% verifying photon indistinguishability [99], enabling high-rate, high-fidelity frequency multiplexed quantum applications.

#### Synthetic dimensions

Photonic synthetic dimensions in the frequency domain [100,101] use discrete optical frequency modes as lattice sites, enabling the simulation of complex systems including high-dimensional and topological physics [102–104]. Cavity-based EO modulation realizes frequency synthetic dimensions, typically with a microwave frequency matching the cavity FSR. Recent work demonstrates a 400 *×*400 mode-space quantum-correlated synthetic crystal using a TFLN microresonator-based quantum frequency comb (Fig. 2f) [85], supporting simulations of quantum random walks, Bloch oscillations, and multilevel Rabi oscillations in the time–frequency correlated space.

### Single-photon detection

Integrated single-photon detectors convert nonclassical optical states into electrical pulses, serving as the primary readout interface for photonic quantum information systems. The scaling and fidelity of protocols such as quantum key distribution (QKD), linear optical quantum computing, and quantum metrology are fundamentally governed by key detector metrics: system detection efficiency (SDE), timing jitter, dark count rate (DCR), and photon-number-resolving (PNR) capability. The overall SDE is mathematically defined by the product of three distinct probabilities:


(9) \begin{eqnarray*}
\eta _{\mathrm{SDE}} = \eta _{\mathrm{c}} \eta _{\mathrm{abs}} \eta _{\mathrm{int}},
\end{eqnarray*}

where $\eta _{\mathrm{c}}$ represents the coupling efficiency from the optical mode to the detector region, $\eta _{\mathrm{abs}}$ is the probability of photon absorption by the superconducting material, and $\eta _{\mathrm{int}}$ denotes the internal quantum efficiency, defining the probability that an absorbed photon triggers a measurable electrical pulse.

Conventional semiconductor single-photon avalanche diodes, including Ge-on-Si and InGaAs/InP variants operating in Geiger mode, provide localized integration paths across near-infrared and telecommunication bands [109–111]. However, these devices are constrained by a rigid trade-off between SDE and noise (typically limiting SDE below 50% at telecom wavelengths), elevated afterpulsing effects, and timing jitter on the order of hundreds of picoseconds. These constraints limit the maximum secure key rate in communication links and restrict the depth of reconfigurable quantum circuits.

SNSPDs address these bottlenecks by utilizing a current-biased superconducting-to-resistive transition [112–114]. SNSPDs routinely exhibit SDE exceeding 90%, sub-10 ps intrinsic timing jitter, and sub-Hz DCR. Crucially, their planar thin-film geometry allows direct integration with nanophotonic waveguides, supporting scalable architectures on Si, Si$_3$N$_4$, and TFLN (Fig. 3a) [15,40]. Two mainstream on-chip integration architectures for SNSPDs have been proposed: direct waveguide integration and resonant cavity-enhanced integration. We discuss these two schemes separately below.

**Figure 3.**
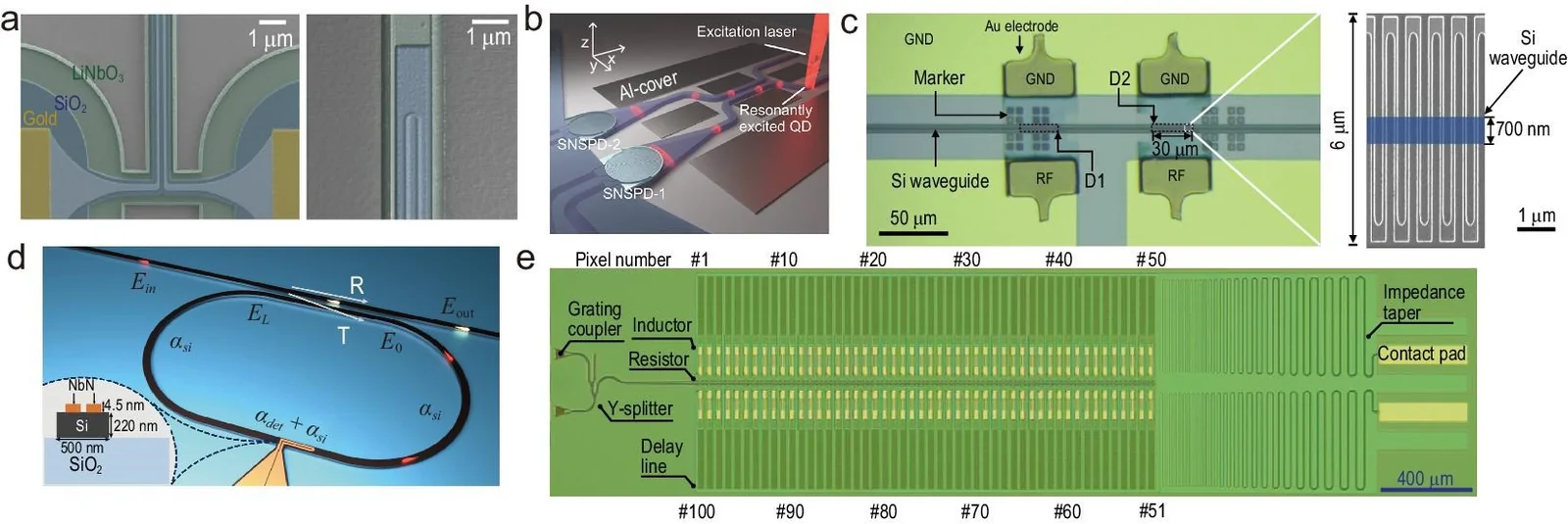
Optoelectronic integrated SNSPDs for single-photon detection. (a) The hairpin-shaped SNSPD design integrated on the LN chip [40]. (b) On-chip integration of quantum dot light source and spiral-shaped SNSPD with tapered waveguide coupling on GaAs substrate [105]. (c) Two comb nanowire detectors placed on the same silicon waveguide for surpassing 99% detection efficiency [106]. (d) Schematic diagram of a SNSPD integrated with a silicon racetrack resonator [107]. (e) The overview optical micrograph of the 100-pixel spatiotemporal-multiplexed SNSPD array-based quasi-PNR detector [108]. Reproduced with permissions from: (a) Ref. [40] under a CC-BY-4.0 license; (b) Ref. [105], copyright: 2018 American Chemical Society; (c) Ref. [106] under a CC-BY-4.0 license; (d) Ref. [107] under a CC-BY-4.0 license; and (e) Ref. [108], copyright: 2023 Springer Nature.

#### Direct waveguide integration detectors

The traveling-wave configuration utilizes evanescent coupling between a nanophotonic waveguide and an overlapping superconducting nanowire, transferring photon absorption from a localized spot to a distributed propagation length. This architecture was established by fabricating NbN nanowires directly on silicon ridge waveguides [115], achieving an absorption coefficient of approximately 1 dB/*μ*m and demonstrating near-unity internal efficiency over tens of micrometers. Subsequent geometric optimizations yielded a 510 ps reset time and a 32 ps timing jitter, validating the throughput capacity for gigahertz-rate quantum processing [116].

For efficient collection from large-mode field on-chip emitters (e.g. quantum dots), spiral nanowire geometries coupled with inverse waveguide tapers were developed to maximize spatial mode overlap (Fig. 3b) [105]. Parallel developments on Si$_3$N$_4$ templates have verified high-efficiency telecom-band operation using NbTiN compounds [117]. More recently, a multi-segment transverse ‘comb’ nanowire array transferred onto a silicon waveguide demonstrated an on-chip SDE of 99.73% at 1550 nm alongside an on-chip self-calibration routine to mitigate coupling-loss uncertainties (Fig. 3c) [106].

#### Resonant cavity-enhanced integration detectors

To circumvent the inherent trade-off between absorption length (which governs kinetic inductance and reset time) and absolute efficiency, cavity-enhanced integration embeds short nanowire segments within high-*Q* optical cavities, such as photonic crystal defect states or microring resonators.

This approach was initially implemented using an asymmetric 2D photonic crystal cavity patterned on a silicon nanobeam [118]. While early iterations suffered from prolonged recovery times (*∼*7 ns) and elevated timing jitter (*∼*55 ps), subsequent optimizations lowered the recovery period to 480 ps while maintaining an on-chip efficiency of *∼*70% [116]. Analytical modeling of silicon racetrack-resonator-integrated SNSPDs quantified the precise timing-jitter penalties associated with cavity round-trips (Fig. 3d) [107,119]. Theoretical calculations indicate that while a 10-*μ*m nanowire section induces a negligible 0.2 ps cavity-contributed jitter, reducing the nanowire length to 1-*μ*m introduces a 10 ps jitter penalty due to multi-pass delay, defining clear boundaries for speed-efficiency co-optimization.

#### Novel structures for multi-pixel arrays and polarization management

Scaling beyond single-channel readouts requires multi-pixel architectures and polarization-insensitive topologies. For highly parallel routing, a multi-tier branching tree waveguide network was deployed to uniformly illuminate a 15-channel integrated SNSPD array, with each pixel operating at internal saturation limits—a configuration highly applicable to neuromorphic processing and multi-photon coincidence counting [120]. Spatial multiplexing has also enabled advanced quasi-PNR schemes capable of resolving high-order photon statistics (Fig. 3e) [108].

In parallel, mitigating nanowire polarization sensitivity remains crucial for fiber-interfaced networks. This limitation was addressed by embedding nanowires within wide GaAs ridge waveguides capable of supporting degenerate TE and TM modes, enabling on-chip second-order correlation measurements under arbitrary input polarizations [121]. Alternatively, a more compact configuration was designed incorporating an on-chip polarization rotator that actively drives TE$_0$ to TM$_0$ mode conversion, enabling near-unity absorption within a compact 5-*μ*m-long nanowire block [122].

Nevertheless, operating waveguide-integrated SNSPDs within complex optical circuits requires systemic cryogenic co-design. Cooling the platform to temperatures below 4 K fundamentally alters the optoelectronic properties of neighboring components. In silicon-based circuits, cryogenic conditions freeze free carriers and suppress the thermo-optic effect, notably impairing the tuning range and energy efficiency of thermal phase shifters [123,124]. For TFLN devices, low-temperature operations induce lattice contraction that reduces the electro-optic coefficients, raises the half-wave voltage ($V_\pi$), and exacerbates photorefractive instabilities. Mitigating these cryogenic effects motivates the exploration of alternative modulation materials, such as graphene electro-absorption modulators [125] and ferroelectric BaTiO$_3$ thin films [126], which retain favorable high-speed switching performance at cryogenic temperatures.

## APPLICATIONS OF INTEGRATED OPTOELECTRONIC QUANTUM CHIPS

### Optoelectronic-driving devices for quantum communication

Quantum communication boasts capabilities that far outperform those of classical communication technologies, and QKD stands out as one of its most prominent applications. Having undergone rapid advancement over the past few decades, QKD is now transitioning toward deployment in commercial fiber-optic networks [134–136] and satellite-based infrastructures [137,138]. Miniaturized, low-cost, and mass-manufacturable high-performance integrated devices form the cornerstone for the scalable deployment of QKD, rendering optoelectronic integrated devices particularly indispensable.

From an implementation standpoint, QKD necessitates high-speed random quantum state preparation, efficient low-noise detection, and dedicated signal post-processing—all of which call for tight integration between electronic control and photonic devices. Correspondingly, the development of photonic integrated circuits (PICs) for QKD has centered on integrated transmitters (incorporating lasers, modulators, and variable optical attenuators) and integrated receivers (equipped with interference circuits, modulators, and detectors), catering to both DV and CV encoding schemes. Silicon (Fig. 4a–i) [127] and TFLN [139,140] platforms are widely adopted in transmitters to achieve high-speed modulation. For the practical generation of weak coherent states (WCSs) based on the decoy state method [141]—a technique that obviates the need for true single-photon sources—laser integration typically requires the incorporation of III–V materials, either through hybrid integration [142,143] or InP-based monolithic integration [13,128,144–146] (Fig. 4a–ii). In contrast, the receiver side prefers low-loss platforms such as Si, Si$_3$N$_4$, SiON, or SiO$_2$ [13,147,148], while also considering the detector integration capability [129,131,133,149,150]. Notably, the recently developed TFLN platform deserves special attention due to its potential to synergize these aforementioned attributes, rendering it a promising candidate for the monolithic integration of QKD transceivers [139,140].

**Figure 4.**
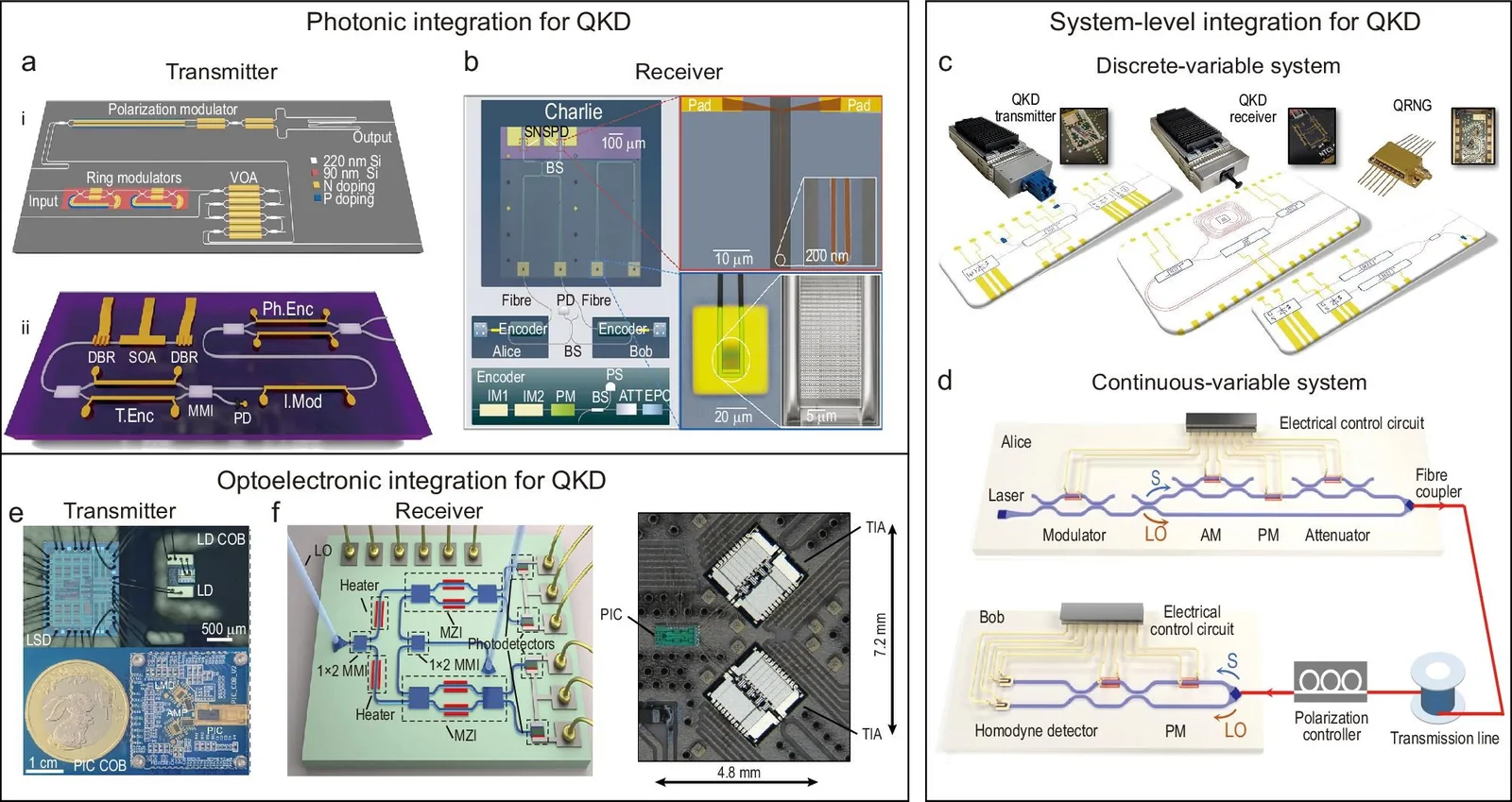
Integrated optoelectronic devices for QKD applications. (a) Photonic integrated transmitters for QKD. (i) The silicon-based transmitter with carrier-dispersion-based high-speed phase modulators and on-chip polarization-encoding modules [127]. (ii) The InP-based transmitter with on-chip lasers, intensity and phase modulators [128]. (b) SNSPD-integrated MDI-QKD receiver. The time-bin encoding Bell state measurement is carried out with a 50:50 beamsplitters and heterogeneous-integrated NbN superconducting nanowires [129]. (c) System-level integration for DV-QKD. The system comprises integrated transmitter, receiver, and QRNG chips, which are packaged using standard approaches as pluggable modules, enabling real-time secure key generation [130]. (d) System-level integration for CV-QKD. Gaussian-modulated CV-QKD was implemented using silicon-based transmitter and receiver chips [131]. An external laser was employed owing to the lack of effective on-chip light-source integration. (e) Optoelectronic fusion integrated transmitter for QKD. The laser and modulator PICs are implemented separately and connected to their respective driver chips via chip-to-chip wire bonding. All chips are co-mounted on a single PCB to enable compact integration and optimized bandwidth performance [132]. (f) Optoelectronic fusion integrated receiver for CV-QKD. It combines a silicon PIC with custom-designed GaAs pHEMT TIAs and could operate at a symbol rate of 10 GBaud [133]. Reproduced with permissions from: (a-i) Ref. [127], copyright: 2016 Optical Society of America; (a-ii) Ref. [128], copyright: 2020 Optica Publishing Group; (b) Ref. [129] under a CC-BY-4.0 license; (c) Ref. [130], copyright: 2021 Springer Nature; (d) Ref. [131], copyright: 2019 Springer Nature; (e) Ref. [132], copyright: 2026 Wiley‑VCH; and (f) Ref. [133], copyright: 2024 Optica Publishing Group.

Point-to-point implementations of superposition-based BB84-type protocols have been demonstrated [127,147,151,152]. In particular, impressive secret key rates of up to 110 Mbps at 10 km have been achieved, with communication distances extended to the 300 km regime [152]. Protocols offering enhanced security have also been realized, most notably measurement-device-independent QKD (MDI-QKD) [153]—featuring integrated transmitters [128,154,155] and SNSPD-integrated receivers [129] (Fig. 4b). A star-type network can be constructed wherein users only need to prepare WCSs, while expensive single-photon detectors are centralized at a shared measurement node. With the improved performance of on-chip lasers [146], it is also feasible to implement more advanced protocols, such as twin-field QKD (TF-QKD) [156], to extend the achievable communication distance. By integrating key components on-chip, Fig. 4c presents a highly practical integrated QKD system based on DV encoding, operating at gigahertz clock rates [130]. The quantum random number generator (QRNG) chips, transmitter chips, and receiver chips are implemented as pluggable modules using standard packaging, with electrical interconnection via high-speed field-programmable gate arrays to enable real-time secure key generation. Notably, only the single-photon detection stage remains not fully integrated on-chip. As illustrated in Fig. 4d, a typical CV-QKD system can fully capitalize on the maturity of silicon photonics technology to achieve on-chip integration of modulators and photodiode detectors [131]. In recent years, substantial efforts have been devoted to advance integrated CV-QKD transmitters [157,158], receivers [133,159], or both [160]. Currently, integrated CV-QKD systems can operate at symbol rates exceeding 10 GBaud [133] and deliver secret key rates in the Mbps regime over metropolitan-area distances [159,160]. Owing to its reliance on room-temperature detectors, intrinsic compatibility with classical optical communication infrastructures, and enhanced tolerance to noise photons, CV-QKD is recognized as a cost-effective solution for practical QKD deployment.

With the continued advancement of PIC technology, the incorporation of electronic integrated circuits (EICs), that is, optoelectronic fusion integration, not only enables higher levels of system integration, but also has the potential to significantly enhance QKD performance at the device level. Typical electronic driving requirements for QKD include pulsed laser driver modules (for phase-randomized pulse generation), multi-level modulator drivers (for decoy-state preparation and signal-state encoding), high-speed low-noise transimpedance amplifiers (TIAs, for CV systems), as well as high-speed analogue-to-digital and digital-to-analog converters required for real-time QRNG integration. Enabled by optoelectronic fusion integration, compact transmitter architectures with optimized bandwidth can be realized [132,161,162]. Figure 4e shows an integrated QKD transmitter using chip-on-board hybrid 2D packaging technology [132]. The laser and modulator driver chips are fabricated based on a SiGe BiCMOS process and are connected to the silicon photonic PIC via direct wire bonding. Compared with schemes relying on discrete electronic control equipment or PCB-based electro–optic interfaces [161,162], this approach further reduces the impact of parasitic capacitance, allowing the system to operate at a high repetition frequency of 3.33 GHz and achieve a secret key rate of 2.82 Mbps over a transmission distance of 100 km using the polarization-encoded BB84 protocol.

Optoelectronic-driven QKD receivers can achieve substantial performance improvements, particularly in systems based on balanced homodyne detection. Although CV-QKD exhibits a strong potential for high secret key rates, early experimental demonstrations using PICs were largely constrained by the electronic bandwidth of the receiver, preventing the full performance of PIC-based devices from being realized [131]. The detection of weak quantum states requires high-gain photoelectric conversion, a regime in which classical high-speed photonic receiver circuits developed for optical communications generally fail to deliver the efficiency and noise performance demanded by quantum applications. Moreover, the use of off-the-shelf or packaged TIAs introduces additional parasitic capacitance, thereby degrading the system bandwidth.

Figure 4f illustrates an optoelectronic-integrated receiver, in which a silicon photonic PIC is wire-bonded to specially designed TIA chips based on 100 nm GaAs pHEMT technology [133]. This architecture enables operation at high symbol rate and supports the implementation of discrete-modulated CV-QKD. Further bandwidth improvements could, in principle, benefit from monolithic optoelectronic integration, which is an advantage of choosing silicon platforms [150]. However, for QKD applications, where practicality is of great concern, the substantial differences in feature sizes between photonic and electronic devices mean that monolithic integration typically relies on compromise process flows, entails increased fabrication complexity and cost, and does not necessarily offer clear advantages over hybrid packaging or 3D integration approaches, in which PICs and EICs can each be fabricated using their respective optimal processes. Similar considerations have also been recognized in the development of classical optoelectronic integrated systems [163].

Optoelectronic integrated devices will further enable advanced quantum communication applications, for example, high-dimensional QKD [164,165], wavelength-division-multiplexed multi-user network [166] and entanglement-based protocols [45,67,69,167–170]. To date, chip-to-chip entanglement distribution [67,167] and quantum teleportation [45,168–170] have been demonstrated, which together with integrated quantum memories [171–173], are expected to form the core building blocks of future quantum-repeater-based networks [174]. Realizing these capabilities requires continued optimization of individual device efficiency and coordinated system-level design, accompanied by extensive (probably cryogenic-compatible) electronic control [37], in order to fully exploit the advantages of integrated optoelectronic platforms.

### Quantum computing

Quantum computing promises transformative capabilities in simulation, optimization, and learning, but realizing scalable advantages with fault-tolerant operation places stringent requirements on phase stability, low-loss interference, fast reconfiguration and feedforward, and high-efficiency measurement. Within the broader landscape of quantum-computing hardware, photonics represents a distinct technical route alongside superconducting circuits, trapped ions, neutral atoms, and semiconductor spin qubits. Its appeal stems from weak optical decoherence, compatibility with long-distance quantum communication, and the ability to encode and multiplex quantum information in path, time, frequency, polarization, and quadrature degrees of freedom. These features make photonics particularly attractive for linear-optical and measurement-based quantum computing, while shifting the main hardware challenges to scalable quantum state generation, optical loss, active switching and feedforward, and efficient measurement. In this context, on-chip optoelectronic integration is particularly compelling because it combines low-loss photonic circuitry with high-speed EO control and scalable on-chip detection, and increasingly interfaces to deterministic sources, offering a plausible path toward large-scale photonic quantum computing [179]. To reflect how these requirements map onto chip capabilities, we broadly organize recent progress into five categories: programmable interferometers, fault-tolerance-relevant resources, fast feedforward control, design for scale, and demonstrative applications.

#### Programmable interferometers

Integrated photonic processors are converging on reconfigurable phase-stable interferometric networks as the computational backbone. Quantum-walk chips exemplify controlled multi-path interference for graph- and walk-based algorithms [180], while programmable nanophotonic processors executing many-photon circuits demonstrate scalable reconfigurability under chip-level stability [10]. Wafer-scale silicon graph photonics extends this fabric to thousands of components, emphasizing manufacturable routes to large scale interferometry, sampling, and entanglement primitives (Fig. 5a) [175]. System focused single-photon processors and ultracompact 3D photonics further highlight deployable control stacks and dense, high-dimensional logic within tightly controlled mode spaces [181,182].

**Figure 5.**
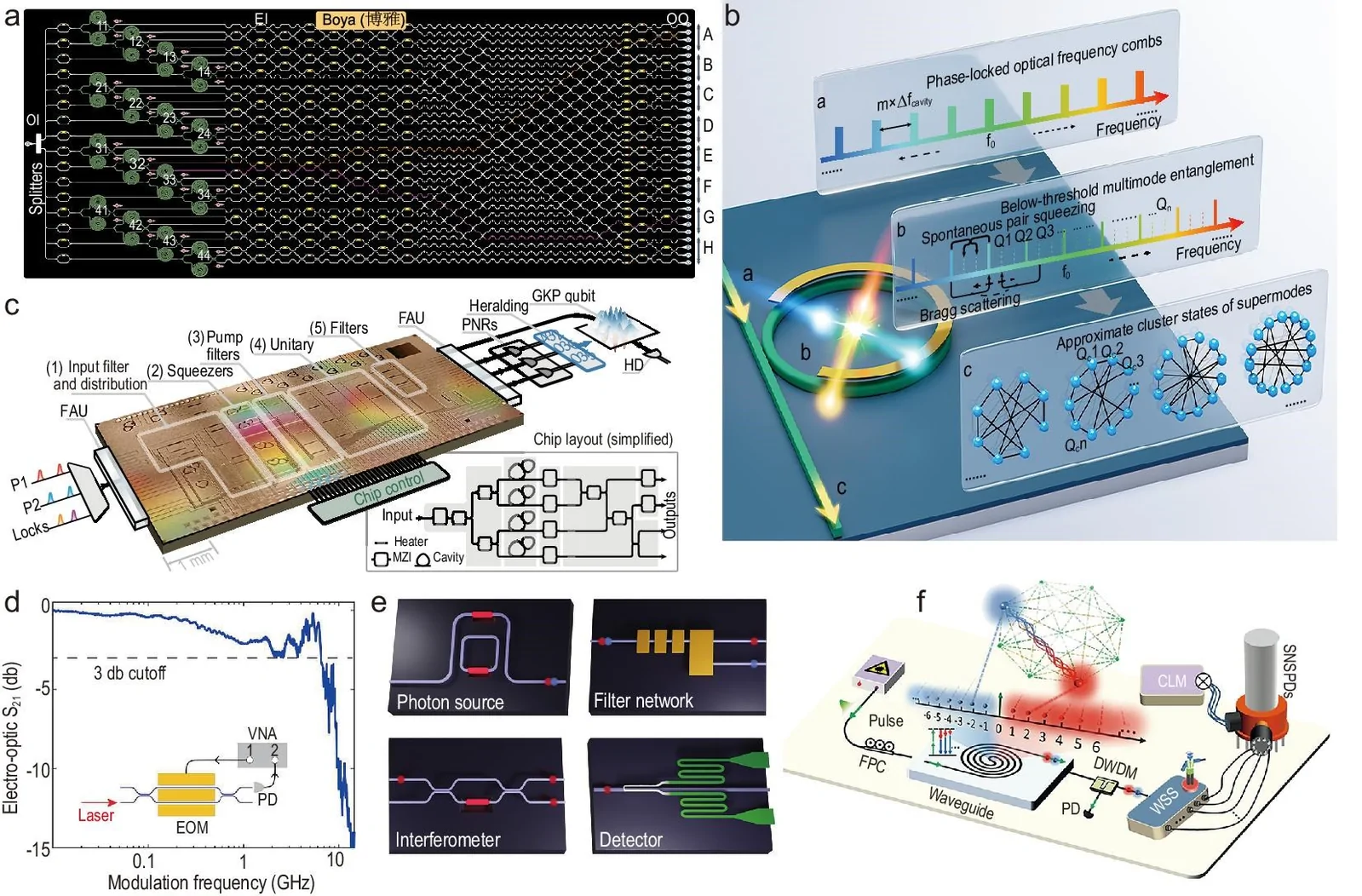
Integrated optoelectronic devices for quantum computing. (a) Programmable interferometers: wafer-scale graph photonics with large-component-count reconfigurable interferometric networks [175]. (b, c) Fault-tolerant resources: chip-scale generation of continuous-variable multipartite entanglement using integrated microcombs [61] and of non-Gaussian Gottesman–Kitaev–Preskill (GKP) resource states for bosonic error correction [176]. (d) Fast feedforward control: GHz-class EO reconfiguration on thin-film lithium niobate enabling low-latency adaptive operations [177]. (e) Design for scale: manufacturable, foundry-compatible photonic integration strategies targeting large-scale systems [15]. (f) Demonstrative application: a frequency-multiplexed photonic processor for perfect-matching sampling [178]. Reproduced with permissions from: (a) Ref. [175], copyright: 2023 Springer Nature; (b) Ref. [61], copyright: 2025 Springer Nature; (c) Ref. [176], copyright: 2025 Springer Nature; (d) Ref. [177] under a CC-BY-4.0 license; (e) Ref. [15], copyright: 2025 Springer Nature; and (f) Ref. [178] under a CC-BY-4.0 license.

#### Fault-tolerant-relevant resources

A complementary thrust targets states and encodings that reduce overhead or directly support fault tolerance. On-chip resource-state engineering can deliver error-protected encodings with algorithm-level benefit [183], and reprogrammable chips have generated hypergraph states as resources for measurement-based quantum computing [184]. In CV platforms, integrated microcombs producing multipartite entanglement provide scalable precursors for cluster-state resources (Fig. 5b) [61], while integrated generation of non-Gaussian GKP states supplies a key bosonic encoding resource for fault-tolerant computation (Fig. 5c) [176].

#### Feedforward control

Measurement-conditioned operation is enabled by high-bandwidth actuation that can respond within relevant temporal or clock-cycle windows. TFLN platforms that demonstrate GHz-class EO reconfiguration provide a critical enabling layer for real-time adaptive circuits and feedforward-compatible control stacks (Fig. 5d) [177].

#### Design for scale

Scaling demands improving the per-operation budget and the system architecture simultaneously. Inverse-designed devices compress universal gate elements to reduce footprint and potentially loss per function [185], while on-chip qudit encodings expand compiled primitives and reduce overhead under fixed loss and stability budgets [186]. At the platform level, modular networking and foundry-compatible approaches emphasize practical routes to large-scale integration with high-efficiency detection and viable interconnect strategies (Fig. 5e) [15,187].

#### Demonstrative applications

Near-term demonstrations leverage photonics-friendly workloads built on large linear-optical transformations, sampling primitives, and analog-style simulation. Notably, Boson sampling provides representative multiphoton interference experiments on integrated photonic chips [188–191]. Waveguide interferometers and on-chip phase modulators form the core hardware for such chip realizations, enabling stable multiphoton interference at small footprint. Integrated layouts eliminate tedious free-space alignment and suppress environmental phase noise. On-chip integrated photonic sources exhibit high brightness and near-perfect photon indistinguishability. Extensions include scattershot configurations using heralded photon sources [192] and Gaussian boson sampling based on squeezed-state inputs [10,193]. Beyond pure sampling tasks, programmable multimode processors have executed Hamiltonian-style simulation workloads that stress coherent control across complex interferometers [194], while large squeezed-state networks advance Gaussian boson sampling for molecular vibronic spectra at growing mode counts [195]. Further specialized photonic algorithms expand this toolbox, including Bernoulli factory constructions [196] and frequency-multiplexed mappings of perfect matchings to multiphoton interference statistics (Fig. 5f) [178].

Another emerging application of integrated photonic quantum processors lies in quantum machine learning, in which programmable interferometers, optical sampling devices, and data-driven optical transformations can be integrated into hybrid quantum–classical learning frameworks. Variational quantum unsampling has been demonstrated on a quantum photonic processor as a learning-based protocol for verification and inference of near-term quantum circuit outputs [197]. Integrated photonic processors have implemented learning-oriented workloads through quantum kernel estimation [198] and adaptive Boson sampling via post-selection [199].

Taken together, these efforts indicate convergence toward integrated platforms that combine programmable interferometric fabrics, fault-tolerance-relevant resources, fast feedforward-capable control, and design for scale principles, positioning optoelectronic integrated chips as leading substrates for photonic quantum computing.

### Quantum metrology

Quantum metrology exploits nonclassical states, quantum interference, and optimized measurement strategies to estimate physical parameters with precision beyond classical shot-noise-limited approaches [204,205]. For integrated optoelectronic chips, the most relevant targets include optical phase, refractive index, sample concentration, magnetic field, temperature, strain, and electric field. Compared with bulk-optical implementations, integrated platforms offer phase stability, compact interferometry, reconfigurable control, scalable routing, and potential co-integration of quantum sources, modulators, detectors, and electronics.

#### Discrete-variable photonic metrology

In DV photonic metrology, path-entangled states, such as NOON states, and adaptive phase-estimation protocols can improve phase sensitivity or phase resolution in the few-photon regime. A reconfigurable integrated waveguide circuit was used to herald two-photon and four-photon path-entangled states for quantum metrology, demonstrating on-chip phase super-resolution with multiphoton entangled states [206]. Silicon photonics has also been used to implement adaptive Bayesian quantum phase estimation, showing that integrated interferometric circuits can support robust phase-estimation protocols relevant to both metrology and quantum algorithms (Fig. 6a) [200].

**Figure 6.**
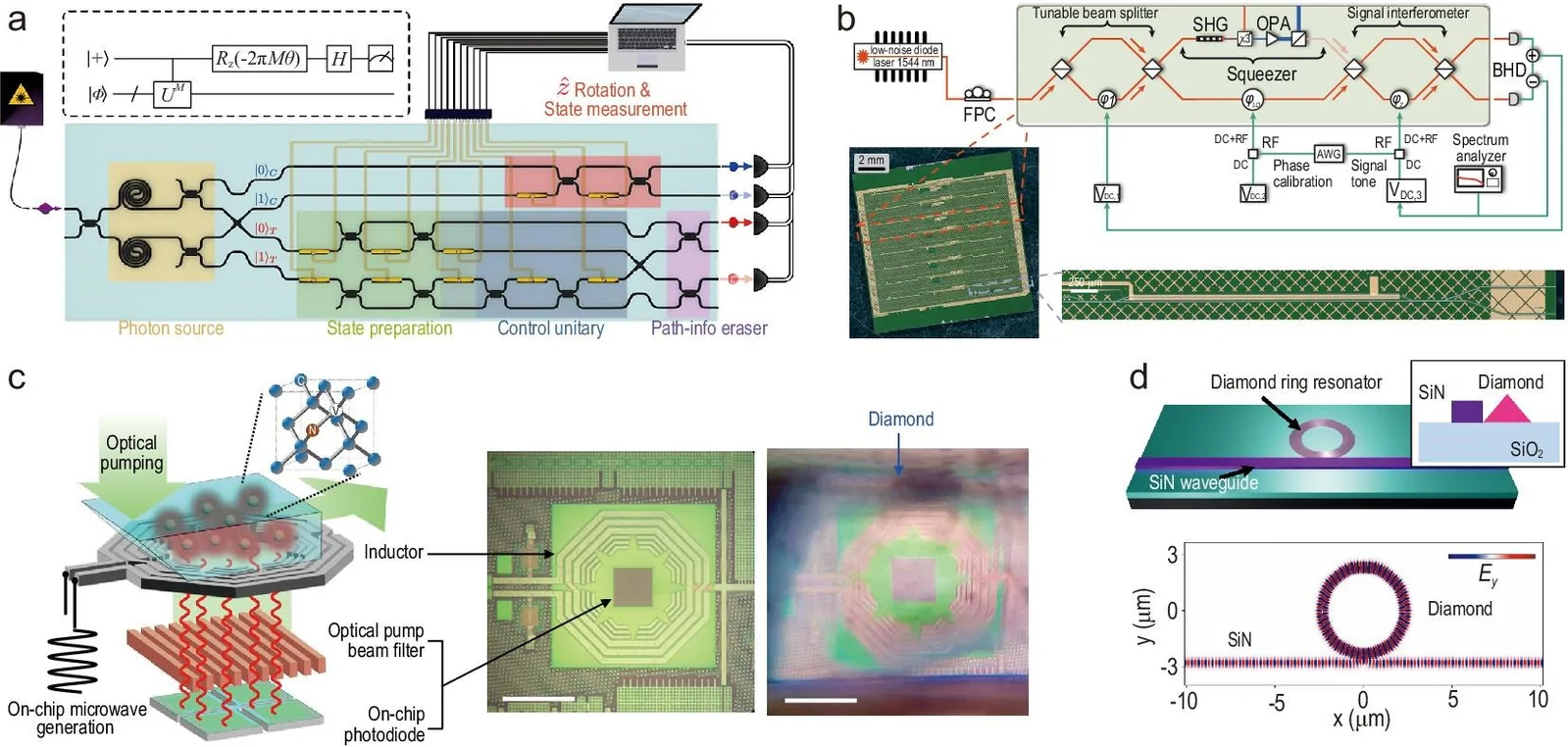
Integrated optoelectronic devices for quantum metrology. (a) Silicon photonic chip for on-chip Bayesian phase estimation [200]. (b) Thin-film lithium niobate photonic chip for integrated squeezed-light phase sensing [201]. (c) A CMOS-integrated quantum sensor based on nitrogen-vacancy centers [202]. (d) Diamond photonic chip based on micro-ring resonators for quantum sensing [203]. Reproduced with permissions from: (a) Ref. [200], copyright: 2017 American Physical Society; (b) Ref. [201] under a CC-BY-4.0 license; (c) Ref. [202], copyright: 2019 Springer Nature; and (d) Ref. [203] under a CC-BY-4.0 license.

#### Continuous-variable photonic metrology

CV quantum metrology uses squeezed states to reduce measurement noise in the optical quadrature relevant to the target parameter [207]. A TFLN photonic integrated circuit has combined squeezed-light generation, electro-optic phase control, and interferometric sensing to realize an integrated quantum optical phase sensor operating beyond the quantum noise limit (Fig. 6b) [201]. On the measurement side, integrated homodyne detectors and photonic-electronic balanced homodyne receivers provide key readout technology for CV metrology, enabling compact quantum-state measurement and high-bandwidth shot-noise-limited detection [208].

#### Solid-state quantum sensors

Integrated optoelectronic technologies are also central to solid-state quantum sensing, especially for nitrogen-vacancy (NV) centers in diamond. A CMOS-integrated NV-center platform combined microwave spin control and photodetection electronics with diamond quantum sensing, enabling compact magnetometry and thermometry (Fig. 6c) [202]. On-chip diamond micro-ring resonators containing NV centers have been used for nanoscale magnetic-field sensing, improving photon utilization and demonstrating high-sensitivity quantum sensing on a photonic chip (Fig. 6d) [203].

Future progress in integrated quantum metrology will require lower optical loss, higher detection efficiency, brighter quantum sources or higher squeezing levels, long-term calibration stability, robust packaging, and tighter integration of sensing interfaces with photonic and electronic control layers.

### Single-photon-level quantum microwave interfaces

The interfaces between the microwave and optical domains are indispensable parts in integrated quantum circuits. Superconducting devices, including qubits, logic circuits, and detectors operating at gigahertz frequencies offer strong controllability but are confined to cryogenic environments with limited connectivity. Optical photons, in contrast, are ideal for low-loss, room-temperature communication. A long-standing goal is therefore a quantum transducer that coherently maps quantum states between microwave and optical photons [94,97,214].

The commonly used scheme for microwave-optical quantum transduction is the cavity EO system, which consists of a superconducting microwave resonator and a doubled-ring coupled-cavity, with the frequency splitting of the optical frequency doublet, corresponding to the symmetric and anti-symmetric supermodes of the photonic molecule, tuned to match the microwave resonance frequency by applying a DC bias through the electrode [96,215,216]. In this scheme, a strong pump in a lower frequency mode $\omega _p$ can upconvert a microwave photon in the frequency mode $\omega _{mw}$ into a signal photon in the higher frequency mode $\omega _s$, described by a Hamiltonian as [216–218]


(10) \begin{eqnarray*}
H &=& \hbar \omega _p a_p^\dagger a_p + \hbar \omega _s a_s^\dagger a_s + \hbar \omega _{mw} a_{mw}^\dagger a_{mw}\\
&& + \hbar g_{EO} \left(a_p^\dagger a_s a_{mw}^\dagger + a_p a_s^\dagger a_\mathrm{mw} \right),
\end{eqnarray*}

where $a_p$, $a_s$, and $a_{mw}$ denote annihilation operators for the optical modes of pump and signal frequency and microwave mode, respectively, and $g_{EO}$ represents vacuum EO interaction strength.

The key figure of merit of cavity EO coherent conversion is quantified by the cooperativity, $C = {4 n_p g_{EO} ^2}/{\kappa _s\kappa _{mw}}$, relying on the intracavity pump photon number $n_p$, EO interaction strength $g_{EO}$, the total energy loss rates for signal $\kappa _s$ and microwave $\kappa _{mw}$. With triply-resonant condition filled ($\omega _p+\omega _{mw}=\omega _s$), the on-chip peak conversion efficiency is given by [96,216,219]


(11) \begin{eqnarray*}
\eta = \frac{\kappa _{s,ex}}{\kappa _s} \frac{\kappa _{mw,ex}}{\kappa _{mw}} \frac{4C}{(1+C)^2}.
\end{eqnarray*}

Here, $\kappa _{s,ex}$ and $\kappa _{mw,ex}$ are the external loss rates for signal and microwave, respectively, and $\eta _{int}=\frac{4C}{(1+C)^2}$ stands for the internal conversion efficiency. The up-to-date superconducting cavity EO conversion in TFLN-superconductor hybrid system reaches 1.02% on-chip efficiency and 15.20% internal efficiency (Fig. 7a) [96]. The conversion efficiency can be further improved by proper cryogenic packaging, high-quality factor cavities, and better optical coupling, which could promise the TFLN-superconductor hybrid EO system to become a highly competitive transduction platform for future quantum network applications.

**Figure 7.**
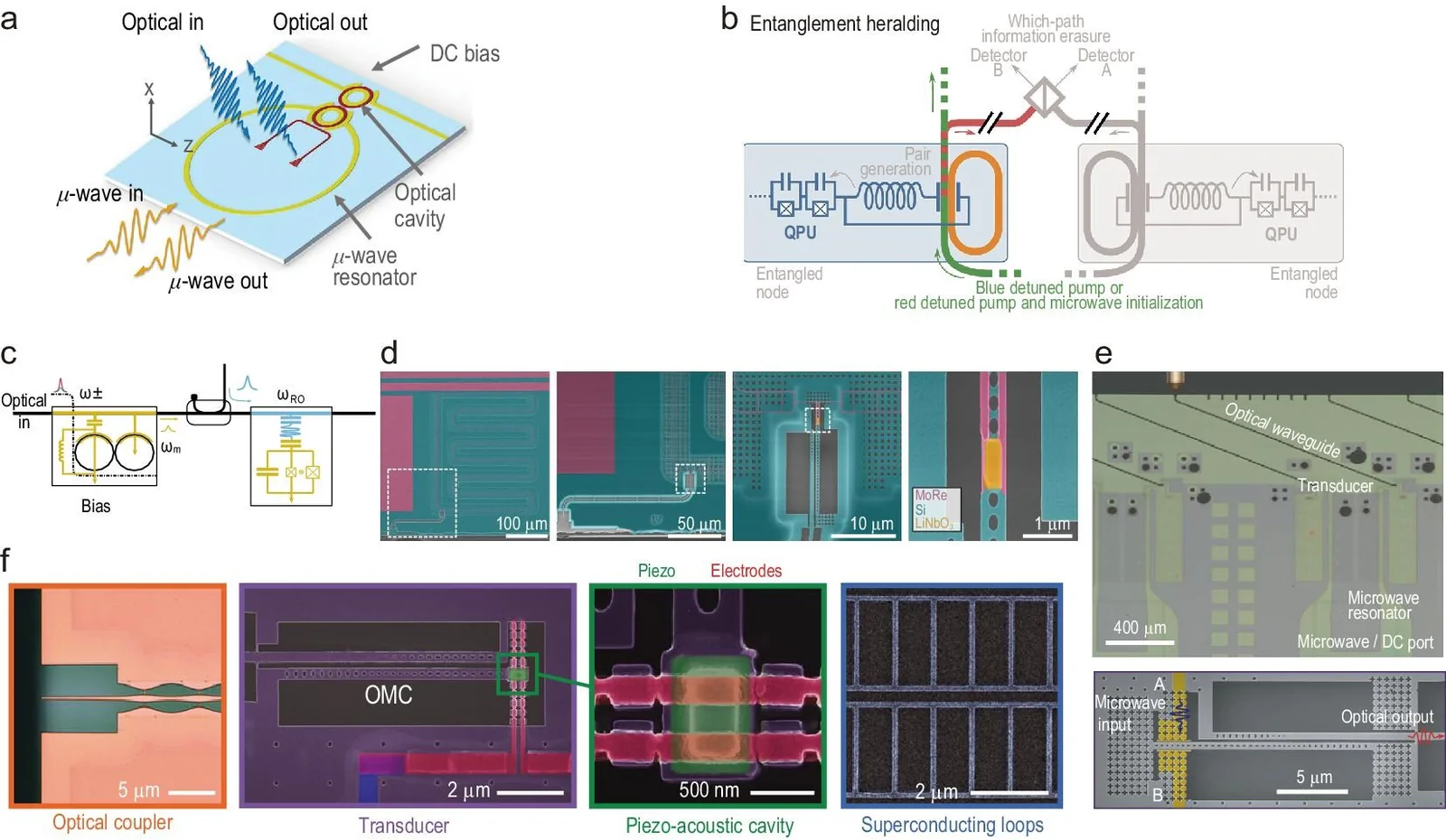
Integrated optoelectronic devices for single-photon-level quantum microwave interfaces. (a) TFLN cavity EO transducer with coupled optical microrings and a superconducting microwave resonator [96]. (b) Optically heralded microwave entanglement based on microwave-optical photon-pair generation [209]. (c) Optical control of a superconducting qubit using a TFLN cavity electro-optic transducer [210]. (d) Scanning electron micrographs of an integrated lithium-niobate-on-silicon piezo-optomechanical transducer [211]. (e) Fabricated silicon electro-optomechanical transducer chip with an array of devices and a representative transducer micrograph [212]. (f) Micrographs of a chip-scale piezo-optomechanical transducer for nonclassical microwave-optical photon-pair generation [213]. Reproduced with permissions from: (a) Ref. [96] under a CC-BY-4.0 license; (b) Ref. [209], copyright: 2021 American Physical Society; (c) Ref. [210], copyright: 2025 Springer Nature; (d) Ref. [211], copyright: 2024 Springer Nature; (e) Ref. [212], copyright: 2025 Springer Nature; and (f) Ref. [213], copyright: 2024 Springer Nature.

Besides the traditional EO transduction which requires high conversion efficiency, a paradigm shift where instead of demanding high-efficiency conversion, one can exploit low-probability but high-fidelity heralded events, has been proposed recently (Fig. 7b) [209]. In this protocol, pairs of optical and microwave photons can be generated in the SPDC process with blue-tuned pump at higher frequency mode. Detection of a single optical photon heralds the creation of its microwave twin, establishing entanglement between spatially separated superconducting nodes. This mechanism is immune to conversion inefficiency and thermal noise, since heralding projects the system onto an entangled state conditioned on photon detection. Hence, it can be further scaled to connect multiple superconducting quantum processing units for multiplexed entanglement necessary for integrated quantum networks. More recently, coherent control of superconducting qubit has been demonstrated using transducer-driven superconducting qubit scheme based on different frequency generation among optical fields and microwave signal, which reported higher conversion efficiency with low added microwave noise (Fig. 7c) [210].

Given the large frequency mismatch between gigahertz microwave photons and telecommunication optical photons, mechanical oscillators are widely employed as an effective intermediary, as they can couple equivalently to fields at vastly different wavelengths [219,220]. Recent experimental breakthroughs have demonstrated integrated optomechanical transducers that enable coherent conversion between microwave and optical photons at near‑quantum-limited noise levels, with notable improvements in conversion efficiency, bandwidth, and scalability (Fig. 7d, e) [211,212]. Platforms based on piezo-optomechanical crystals have realized nonclassical photon pair generation between microwave and optical domains, directly verifying the quantum nature of the transduction process (Fig. 7f) [213]. Theoretical designs further explore diamond optomechanical cavities coupled to color centers [221], promising higher intrinsic efficiency and better coherence. These developments establish optomechanical cavity interfaces as another viable approach for bridging superconducting quantum processors and optical quantum networks.

## OUTLOOK

Integrated optoelectronic quantum chips have advanced dramatically in the past decade, evolving from proof-of-concept demonstrations to sophisticated systems capable of generating, manipulating, and detecting quantum states of light with high fidelity and scalability. As we look to the future, several key challenges and opportunities will shape the next generation of integrated quantum photonic technologies.

While significant progress has been made in integrating individual components—such as quantum light sources [34,57], EO modulators [89,222], and SNSPDs [40,106]—the full co-integration of all these elements on a single chip remains a formidable goal. Future efforts must address material incompatibilities, thermal management, and optical coupling losses in heterogeneous platforms [33,34]. The development of wafer-scale, foundry-compatible processes [15], and advanced packaging techniques [42] will be critical for moving from laboratory prototypes to manufacturable and scalable quantum photonic systems.

Key performance metrics such as photon indistinguishability, detection efficiency, and modulation bandwidth must continue to improve to meet the demands of fault-tolerant quantum computation and long-distance quantum communication. For quantum light sources, deterministic and tunable emitters based on quantum dots [36] or molecules [33] offer promising paths toward high-purity, on-demand photon generation. In parallel, the integration of high-speed EO modulators on TFLN [83,84] and silicon [20] platforms will enable faster reconfiguration and feedforward control essential for dynamic quantum protocols [177]. Looking ahead, we anticipate the emergence of novel optoelectronic effects and materials that may offer superior performance for specific quantum applications. For instance, the integration of two-dimensional materials with strong light-matter interaction could enable compact, low-power quantum light modulators, while advances in piezoelectric materials may provide new mechanisms for tuning quantum emitters with high precision and low thermal noise.

In quantum communication, the transition from laboratory demonstrations to field-deployable systems will benefit from further optoelectronic integration. Current integrated QKD systems already combine modulators, passive circuits, and in some cases on-chip lasers [13,128,139]. The next generation of systems should aim for full transmitter–receiver integration with embedded control electronics, enabling plug-and-play quantum communication modules. For long-distance quantum networks, the integration of quantum memories based on rare-earth-doped materials [173] or color centers [172] with tunable couplers and frequency converters will be essential. Moreover, the development of cryogenically compatible optoelectronic interfaces will facilitate the connection between photonic quantum links and superconducting quantum processors [210]. Beyond point-to-point links, future quantum networks will require advanced routing and switching capabilities. The integration of programmable optical switches with quantum-state-preserving characteristics could enable reconfigurable quantum network topologies, while the development of quantum repeaters with integrated memory-control interfaces will be crucial for extending quantum communication to global scales.

For quantum computing applications, optoelectronic integration must support both the high precision required for fault-tolerant operations and the high speed needed for practical computational throughput. Programmable photonic processors with thousands of components [175] will require advanced calibration and control schemes that leverage on-chip photodetectors and electronic feedback loops. The integration of photon-number-resolving detectors [108] will enable more sophisticated measurement-based quantum computing protocols, while fast EO switches will facilitate resource-state generation through temporal multiplexing. As photonic quantum computing chips continue to grow rapidly in size and complexity, compact electronic control units are urgently required to enable efficient, scalable manipulation of numerous on-chip functional components. Further research should therefore explore the collaborative integration of photonic quantum chips with traditional ASIC chips via hybrid packaging and heterogeneous interconnection. This approach helps establish a versatile and scalable control architecture, lower overall system overhead, and advance the large-scale deployment and practical implementation of integrated quantum photonic systems.

Beyond traditional quantum information applications, optoelectronic quantum chips open avenues for hybrid quantum-classical systems. Quantum sensing applications could leverage integrated squeezed light sources [49] and high-efficiency detectors for beyond-classical sensitivity in measurements of physical quantities. Quantum simulators based on synthetic dimensions [85] or CV cluster states [61] could exploit electronic programmability to explore complex physical phenomena. Furthermore, the integration of machine learning accelerators with quantum photonic processors could enable adaptive quantum experiments and efficient characterization of large-scale quantum devices.

The convergence of integrated photonics, electronics, and quantum optics has created a powerful platform for quantum information processing. As fabrication techniques mature and system integration improves, optoelectronic quantum chips will transition from specialized laboratory instruments to deployable technologies. By addressing the challenges of scalability, performance, and interoperability, these advances could lead to the realization of large-scale quantum networks, fault-tolerant quantum processors, and a new generation of quantum-enhanced devices—ushering in an era of practical quantum technologies.
